# *Reynoutria* Rhizomes as a Natural Source of SARS-CoV-2 Mpro Inhibitors–Molecular Docking and In Vitro Study

**DOI:** 10.3390/ph14080742

**Published:** 2021-07-29

**Authors:** Izabela Nawrot-Hadzik, Mikolaj Zmudzinski, Adam Matkowski, Robert Preissner, Małgorzata Kęsik-Brodacka, Jakub Hadzik, Marcin Drag, Renata Abel

**Affiliations:** 1 Department of Pharmaceutical Biology and Botany, Wroclaw Medical University, 50-556 Wroclaw, Poland; bbsekret@umed.wroc.pl (A.M.); renata.abel@charite.de (R.A.); 2 Department of Chemical Biology and Bioimaging, Wroclaw University of Science and Technology, 50-370 Wroclaw, Poland; mikolaj.zmudzinski@pwr.edu.pl (M.Z.); marcin.drag@pwr.edu.pl (M.D.); 3 Structural Bioinformatics Group, Institute for Physiology, Charité–University Medicine Berlin, 10115 Berlin, Germany; robert.preissner@charite.de; 4 Research Network Łukasiewicz—Institute of Biotechnology and Antibiotics, Starościńska 5, 02-516 Warsaw, Poland; kesikm@iba.waw.pl; 5 National Medicines Institute, ul. Chełmska 30/34, 00-725 Warszawa, Poland; 6 Department of Dental Surgery, Wroclaw Medical University, 50-425 Wroclaw, Poland; jakub.hadzik@umed.wroc.pl

**Keywords:** *Polygoni cuspidati rhizoma*, *Reynoutria sachalinensis*, vanicoside, proanthocyanidins, COVID-19 (Coronavirus disease 2019)

## Abstract

More than a year has passed since the world began to fight the novel severe acute respiratory syndrome coronavirus 2 (SARS-CoV-2) responsible for the Coronavirus disease 2019 (COVID-19) pandemic, and still it spreads around the world, mutating at the same time. One of the sources of compounds with potential antiviral activity is Traditional Chinese Medicinal (TCM) plants used in China in the supportive treatment of COVID-19. *Reynoutria japonica* is important part of the Shu Feng Jie Du Granule/Capsule-TCM herbal formula, recommended by China Food and Drug Administration (CFDA) for treatment of patients with H1N1- and H5N9-induced acute lung injury and is also used in China to treat COVID-19, mainly combined with other remedies. In our study, 25 compounds from rhizomes of *R. japonica* and *Reynoutria sachalinensis* (related species), were docked into the binding site of SARS-CoV-2 main protease. Next, 11 of them (vanicoside A, vanicoside B, resveratrol, piceid, emodin, epicatechin, epicatechin gallate, epigallocatechin gallate, procyanidin B2, procyanidin C1, procyanidin B2 3,3’-di-*O*-gallate) as well as extracts and fractions from rhizomes of *R. japonica* and *R. sachalinensis* were tested in vitro using a fluorescent peptide substrate. Among the tested phytochemicals the best results were achieved for vanicoside A and vanicoside B with moderate inhibition of SARS-CoV-2 Mpro, IC_50_ = 23.10 µM and 43.59 µM, respectively. The butanol fractions of plants showed the strongest inhibition of SARS-CoV-2 Mpro (IC_50_ = 4.031 µg/mL for *R. sachalinensis* and IC_50_ = 7.877 µg/mL for *R. japonica*). As the main constituents of butanol fractions, besides the phenylpropanoid disaccharide esters (e.g., vanicosides), are highly polymerized procyanidins, we suppose that they could be responsible for their strong inhibitory properties. As inhibition of SARS-CoV-2 main protease could prevent the replication of the virus our research provides data that may explain the beneficial effects of *R. japonica* on COVID-19 and identify the most active compounds worthy of more extensive research.

## 1. Introduction

The severe acute respiratory syndrome coronavirus 2 (SARS-CoV-2), responsible for Coronavirus disease 2019 (COVID-19) pandemic, was first reported in December 2019 in Wuhan, China [1,2] Since then, over 180 million confirmed cases and more than 3.8 million fatalities caused by COVID-19 disease were reported worldwide (Johns Hopkins University database: https://coronavirus.jhu.edu/data, accessed on 27 July 2021). SARS-CoV-2 belongs to the *Coronaviridae* family, the same family as previously known coronaviruses such as SARS-CoV, responsible for Severe Acute Respiratory Syndrome and MERS-CoV, which caused Middle East Respiratory Syndrome [3]. The most common symptoms of the infection are: fever, dry cough, shortness of breath, fatigue, diarrhea, rash on skin, or loss of taste and smell. A few of the symptoms usually occurred in combination, depending on the immune system of the individual [4,5]. Not all the infected people are showing symptoms of the disease, which is why extraordinary measures such as social distancing, wearing masks and frequent testing were imposed by authorities around the world. 

Task forces from all over the world are working on designing vaccines and suggesting drugs to fight COVID-19. Vaccine development has given us hope to take control of the pandemic, however because of problems with availability and distribution of vaccines, lack of knowledge for how long the vaccine-induced immunity, as well as the virus’s ability to mutate, there is an urgent need to find effective drugs for this disease. Different computational methods and drug repurposing techniques such as molecular docking, molecular dynamics, or AI (Artificial Intelligence) approaches [6,7,8,9] were used for drugs against SARS-CoV-2 searching. Many of the suggested drugs are currently in clinical trials [10]. There are also many examples of compounds suggested to be potential inhibitors of main coronavirus targets found in different databases such as the ZINC database (ref ZINC 1bilion) or natural product databases or TCM databases [11,12,13].

In this study, compounds and extracts from underground parts (rhizomes) of two medicinal plants—*Reynoutria japonica* Houtt. (syn. *Fallopia japonica* (Houtt.) Ronse Decr., *Polygonum cuspidatum* Sieb. & Zucc.) and *Reynoutria sachalinensis* (F.Schmidt) Nakai (Polygonaceae) (syn. *Fallopia sachalinensis* (F.Schmidt) Ronse Decr., *Polygonum sachalinense* F.Schmidt) were investigated for inhibition of one of the nonstructural proteins of the virus which is the main protease (Mpro, also called 3CLpro). Inhibition of this enzyme could prevent the replication of the virus. Besides the main protease, SARS-CoV-2 virus encodes other nonstructural proteins such as: papain-like protease (PLpro), RNA-dependent RNA polymerase (RdPp), a helicase–triphosphatase, an exoribonuclease, an endonuclease, and N7- and 2′*O*-methyltransferases and four structural proteins: spike, envelope, membrane, and nucleocapsid [14,15]. Many natural products from different medicinal plants have potential antiviral activity against coronaviruses such as SARS-CoV-2, as well as SARS-CoV or other viruses [16,17,18,19,20,21,22].

*R. japonica* is a well-known herb which has rhizomes (Huzhang in Chinese) that are used in China and Japan to treat various inflammatory diseases, infections, skin diseases, and hyperlipidemia [23]. Huzhang is part of the Shu Feng Jie Du Granule/Capsule-TCM herbal formula mainly used in China for the treatment of acute upper respiratory tract infections such as the flu, swelling, and pain in the throat and others [24]. Based on the Shu Feng Jie Du Capsule antiviral effect, the China Food and Drug Administration (CFDA) has recommended use of it for the treatment of patients with H1N1- and H5N9-induced acute lung injury [25]. In 2020, Shu Feng Jie Du Granule/Capsule was used to treat COVID-19, combined with other remedies or alone [26,27]. Rhizomes of *R. japonica* are rich sources of active phytochemicals such as stilbenes, anthraquinones, flavanols, proanthocyanidins, and phenylpropanoid disaccharide esters. The latter are present in greater quantities in the related *R. sachalinensis*, also included in this study. Stilbenes exhibit diverse biological activities such as antioxidative, antitumoral, anti-inflammatory, and antiviral properties [28]. Moreover, recent molecular docking studies showed that stilbenes in general and resveratrol in particular can be promising anti-COVID-19 drug candidates, acting as an inhibitor of the ACE2 receptor and preventing the S1: ACE2 complex formation and entry of the virus into host cells [28]. Emodin, an anthraquinone, blocked the interaction of SARS-CoV S protein and ACE2 in a dose-dependent manner with an IC_50_ of 200 µM as well as inhibited the infectivity of S protein-pseudotyped retrovirus to Vero E6 cells [29]. Also, simple flavanols like epicatechin or epigallocatechin gallate were reported to inhibit angiotensin-converting enzyme activity [30]. Molecular docking and dynamics studies carried out by Maroli et al. [31] showed that procyanidins could be a potential inhibitor of SARS-CoV-2 Mpro as well as ACE2 or spike protein. Phenylpropanoid disaccharide esters present in *Reynoutria* species with a predominant amount of vanicoside B and A [32] are still under-studied chemicals in terms of their biological activity. Their antioxidant and cytotoxic activity against some human tumor cell lines [33,34] as well as their activity as acetylcholinesterase and β-glucosidase inhibitors [35] have been revealed. So far, no studies have been conducted to check their antiviral activity.

For this study we pulled 25 compounds belonging to 5 different classes of phytochemicals from *Reynoutria japonica* and/or *R. sachalinensis.* They were: stilbenes: resveratroloside, piceatannol, piceatannol glucoside, piceid, resveratrol; anthraquinones: emodin, emodin 8-glucoside, emodin bianthrone, physcion; phenylpropanoid disaccharide esters: vanicoside A, vanicoside B, vanicoside C, hydropiperoside, lapathoside A, lapathoside C, tatariside B; flavan-3-ols and procyanidins: epicatechin, epicatechin gallate, epigallocatechin gallate, procyanidin B2, procyanidin B2 3’-*O*-gallate, procyanidin B2 3,3’-di-*O*-gallate, procyanidin C1, procyanidin C1 3’,3’’-di-*O*-gallate, cinnamtannin A2. First, molecular docking was performed on those compounds to evaluate them as potential inhibitors against SARS-CoV-2 Mpro. Consequently, the shortlisted compounds as well as the extracts and fractions from the *R. japonica* and *R. sachalinensis* rhizomes were tested in vitro by means of the spectrofluorimetric assay using recombinant enzyme.

## 2. Results

### 2.1. Molecular Docking Studies

**Figure 1:**
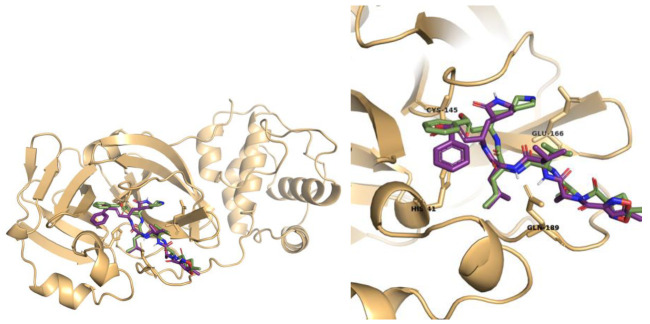
Superimposition of the re-docked (purple) and crystallographic (green) N3 ligand poses.

25 compounds (Figure S1), belonging to five different phytochemical classes that are active compounds of *R. japonica* and *R. sachalinensis,* were docked into the binding site of SARS-CoV-2 main protease. First, poses of re-docked N3 ligand were analyzed and root mean square deviation (RMSD) between re-docked poses and crystallographic N3 ligand was calculated. Also, 3D visualization of the superimposed crystallographic ligand with the best docked pose (RMSD = 1.6369 Å) of N3 ligand is shown below (Figure 1).

**Figure 2:**
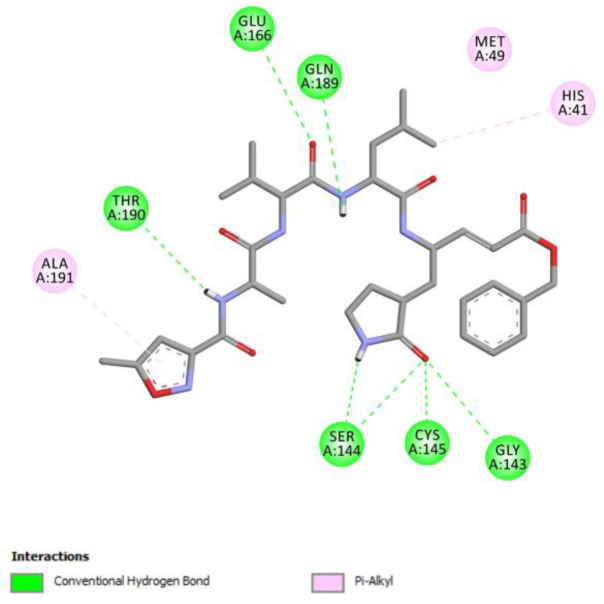
2D visualization of ligand interactions of re-docked N3 ligand into the binding site of SARS-CoV-2 main protease.

RMSD analyses and analysis of interactions showed that N3 ligand was successfully re-docked and that hydrogen bonds with such residues as Gly 143, Ser144, Cys145, Glu166, Gln189, Thr190, and Pi-Alkyl interactions with His41 and Ala191 were observed (Figure 2).

Analysis of results of all docked compounds were based on the visual inspection of interactions with catalytic residues site of Mpro (Cys145 and His41) as well as comparison of interactions of re-docked ligand (N3). Below (Table 1 and Table 2), we are presenting 2D interactions diagrams of those 11 compounds, which were also evaluated in vitro as potential inhibitors of Mpro. Conventional hydrogen bonds and also Pi-Pi interactions are listed in the Table 1 below each compound and common interactions with the N3 ligand are marked in bold. Additional docking results are presented in the Supplementary table (Table S1). The choice of compounds for in vitro study was based not only on the best docking results but was also determined by the availability and quantity of isolated compounds. GOLD docking scores of best poses of the compounds are presented in the Supplementary table (Table S2).

Based on the interaction analyses best potential candidates for Mpro inhibitors are presented in Table 1. Table 2 includes compounds which are not supposed to be good inhibitors, but still were evaluated in vitro study to confirm this assumption.

Based on analyses of 11 main compounds we assumed that phenylpropanoid disaccharide esters such as vanicoside A and vanicoside B or procyanidins such as procyanidin B2 3,3’-di-*O*-gallate and procyanidin C1 as well as emodin are potential inhibitors of Mpro. In the case of all those five compounds (Table 1) the interactions with the catalytic residues Cys145 and His41 were observed. Additionally, hydrogen bonds with such residues as Gly143, Glu166, Gln189, His163, or Thr190 were formed. Interactions with those residues were also present in case of re-docked N3 ligand. Epicatechin, (−)-epigallocatechin gallate or epicatechin gallate are probably not as good Mpro inhibitors, because of lack of the interactions with catalytic residues Cys145 or His41. Resveratrol, piceid, and procyanidin B2 were also classified as poor candidates for Mpro inhibitors. In those cases the interactions with Cys145 or His41 were observed, but hydrogen bond interactions with other residues, common with co-crystalized ligand, were absent (Table 2). Further, the analyses of GOLD docking scores (Table S2) shows that in the case of compounds presented in Table 1, their scores are higher (Goldscore.Fitness > 90) than in the case of compounds presented in Table 2, and higher fitness scores indicate better docking results.

**Figure 3:**
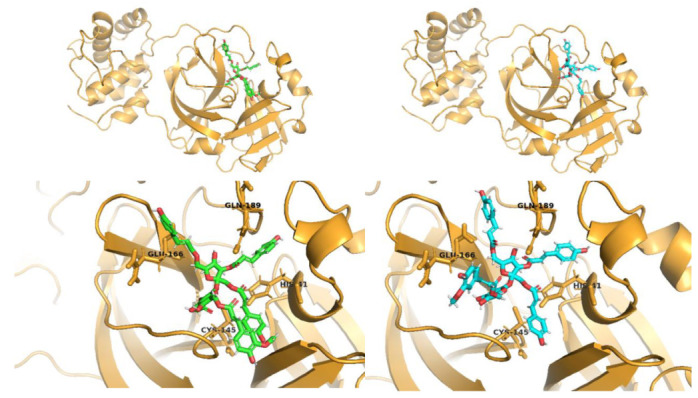
3D visualization of best scored compounds: Vanicoside A (images on the left—green), Vanicoside B (images on the right—cyan).

Analyses of interactions with Mpro residues of additional 14 compounds, which are included in supplementary table (Table S1), but were not tested in vitro, shows that procyanidins such as procyanidin C1 3′,3″-di-*O*-gallate and cinnamtannin A2 as well as phenylpropanoid disaccharide esters such as hydropiperoside, tatariside B, lapathoside C or vanicoside C, and emodin bioanthrone could all be promising candidates for Mpro inhibitors and considered for further in vitro testing. However, these compounds occur in the studied plant material in minor amounts only and as such, they were unavailable in amounts sufficient for thorough pharmacological investigations. GOLD docking scores (Table S2) in case of those compounds are also higher than in case of the remaining seven compounds presented in Table S1, with the exception of emodin bioanthrone, where docking score is much lower. Compounds such as resveratroloside and piceatannol, piceatannol glucoside, procyanidin B23′-*O*-gallate, physcion, emodin-8-glucoside, and lapathoside A are assumed to be worse candidates either because of low number of hydrogen bonds, which could cause the instability of complexes with protease or because of lack of interaction with both catalytic residues Cys154 and His41. Moreover, GOLD fitness scores are also lower (<80) in all those cases. In addition to 2D interaction visualizations presented above, the 3D visualizations of two highest scored compounds: Vanicosides (A and B) in complex with receptor were generated and shown in Figure 3.

### 2.2. Inhibition of SARS-CoV-2 Mpro Enzyme-In Vitro Study

**Figure 4:**
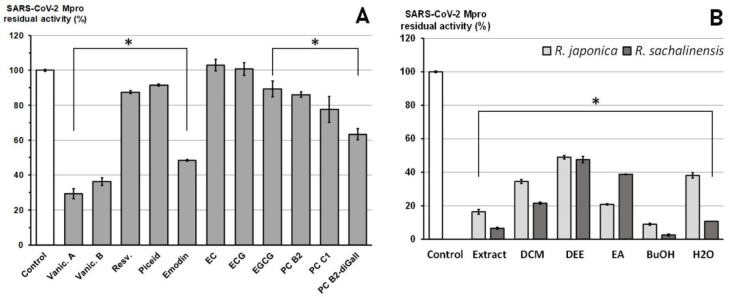
Screening for SARS-CoV-2 Mpro inhibitors. Inhibitors (I), with final concentration equal to 100 µM for individual compounds (**A**) and 50 µg/mL for extracts and fractions (**B**) The results were presented as SARS-CoV-2 Mpro residual activity (%), in relation to control without inhibitor. Error bars shown in this figure are means ± SD for *n* ≥ 3. * Statistically significant at *p* ≤ 0.05 compared to control. Abbreviations: Vanic.—vanicoside, Resv.—resveratrol, EC-epicatechin, ECG—epicatechin gallate, EGCG—epigallocatechin gallate, PC—procyanidin, di-Gall—3,3′-*O*-digallate, DCM—dichloromethane, DEE—diethyl ether, EA—ethyl acetate, BuOH—*n*-butanol.

Eleven compounds (Figure 4A, Table S3) and 12 plant extracts and fractions (Figure 4B, Table S4) were studied in vitro against recombinant SARS-CoV-2 Mpro in spectrofluorimetric assay. During the experiment we used a novel fluorescent peptide substrate (QS1, Ac-Abu-Tle-Leu-Gln-ACC) [36].

**Figure 5:**
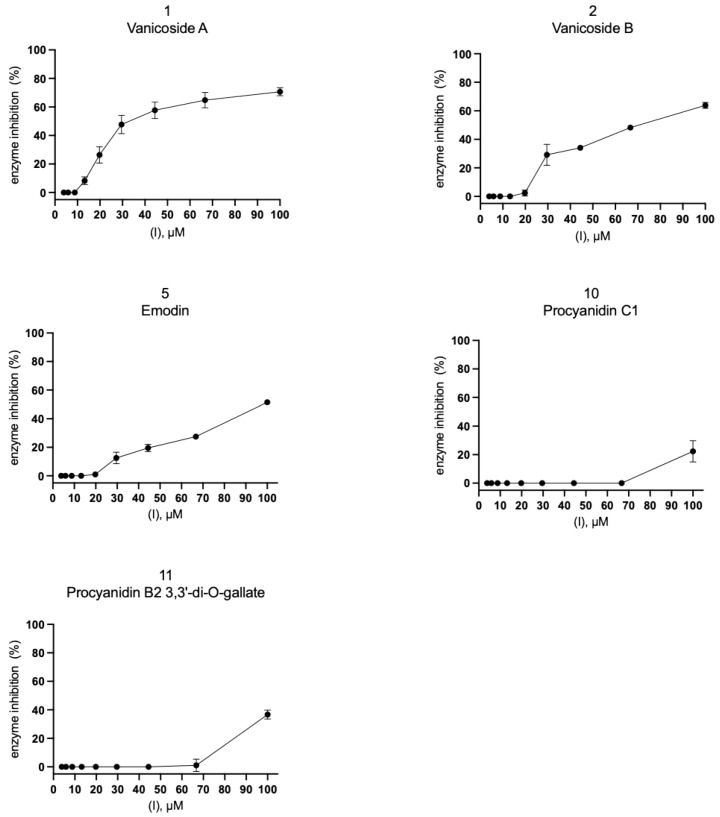
SARS-CoV-2 Mpro activity in serial dilution of compounds. The results were presented as SARS-CoV-2 Mpro inhibition (%).

**Figure 6:**
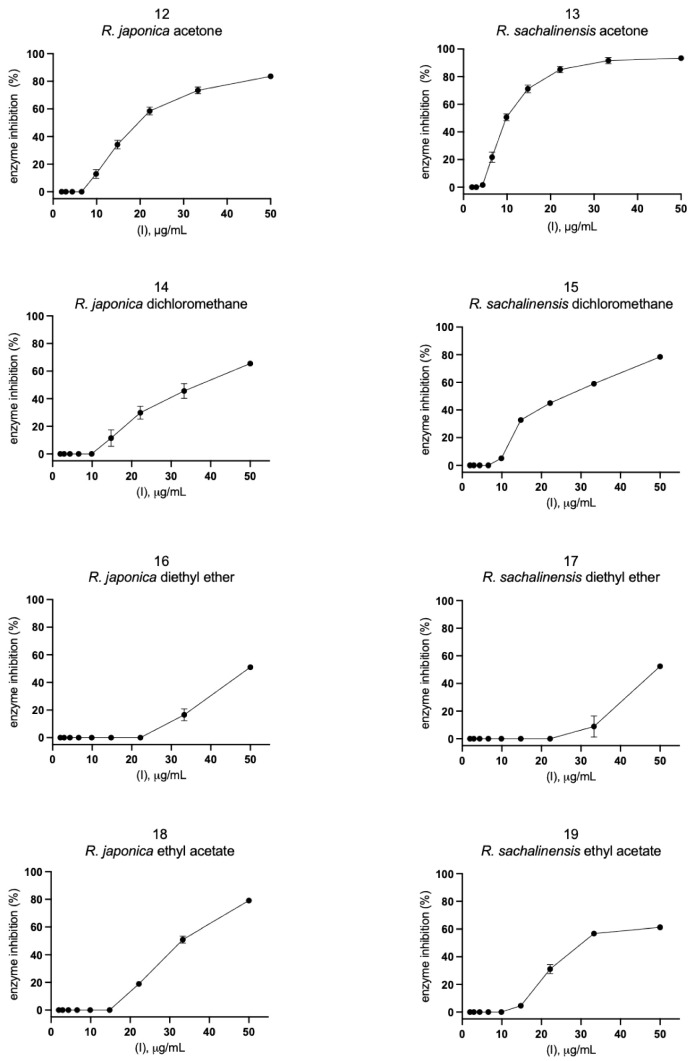
SARS-CoV-2 Mpro activity in serial dilution of extracts and fractions. The results were presented as SARS-CoV-2 Mpro inhibition (%).

**Figure 7:**
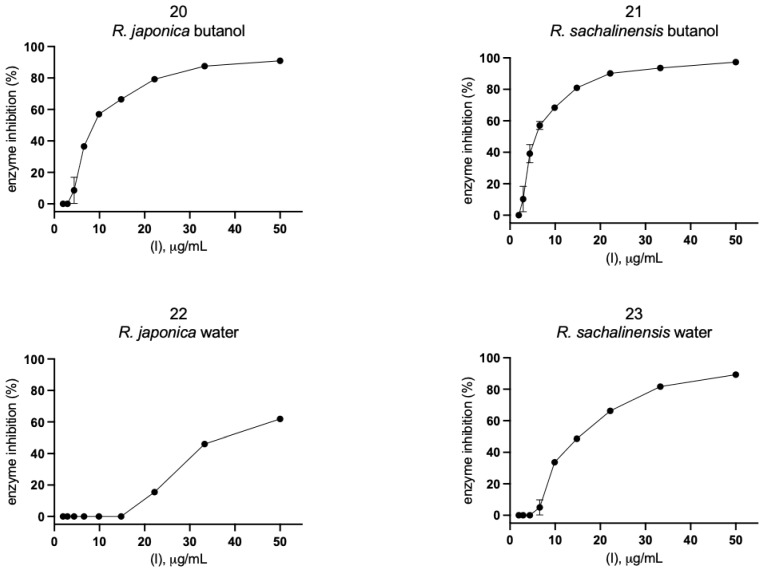
SARS-CoV-2 Mpro activity in serial dilution of extracts and fractions. The results were presented as SARS-CoV-2 Mpro inhibition (%).

**Figure 8:**
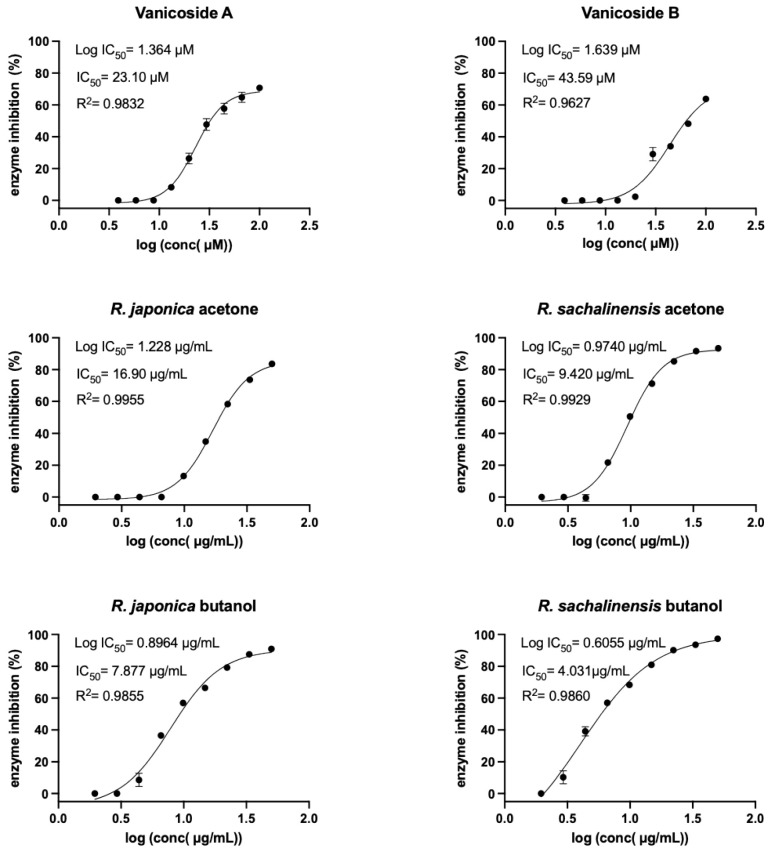
SARS-CoV-2 Mpro activity in serial dilution of the most potent inhibitors. Log IC_50_, IC_50_ and R^2^ were calculated for each sample.

The choice of *R. japonica* and *R. sachalinensis* extracts and fractions was dictated by the promising docking results with compounds present in these plants. The detailed phytochemical composition of the tested extracts and fractions were presented in our earlier studies [33]. Nine out of 11 tested compounds with final concentration of 100 µM inhibited SARS-CoV-2 Mpro enzyme significantly, whereas five of them displayed over 20% inhibition during the screening and were selected for further analysis. Three compounds: vanicoside A, vanicoside B, and emodin revealed over 50% inhibition of the enzyme. All of the studied extracts and fractions with the final concentration of 50 µg/mL significantly inhibited SARS-CoV-2 Mpro, displaying over 50% inhibition of the enzyme, and were selected for further analysis. In the next step, determination of enzyme inhibition in serial dilutions of the selected compounds and extracts/fractions was defined (Tables S5–S8, Figure 5, Figure 6, Figure 7 and Figure 8). Three compounds—vanicoside A, vanicoside B, and emodin—showed significant inhibition of SARS-CoV-2 Mpro, also at low concentrations (starting at 13.2 µM), while the remaining two compounds—procyanidin C1, procyanidin B2 3,3’-di-*O*-gallate—showed significant inhibition only at the highest concentration—100 µM. The Log IC_50_, IC_50_, and R^2^ were calculated for vanicoside A (IC_50_ = 23.10 µM) and vanicoside B (IC_50_ = 43.59 µM), (Figure 8). Among extracts, stronger inhibition of SARS-CoV-2 Mpro was seen for *R. sachalinensis* acetone extract than for *R. japonica* acetone extract, however both achieved low IC_50_ = 9.42 µg/mL and 16.90 µg/mL, respectively (Figure 8). All fractions (dichloromethane (CH_2_Cl_2_), diethyl ether (Et_2_O), ethyl acetate (AcOEt), butanol (*n*-BuOH) and water) were obtained during the fractionation process of acetone extracts [33]. Among the fractions, only butanol fractions showed stronger enzyme inhibition than the corresponding acetone extracts (Figure 6 and Figure 7, Table S8). The IC_50_ was 4.031 µg/mL for *R. sachalinensis* butanol fraction and 7.877 µg/mL for *R. japonica* butanol fraction (Figure 8). It is supposed that compounds present in the butanol fractions are responsible for observed strong inhibition of main protease by *Reynoutria* extracts.

## 3. Discussion

The 25 compounds, previously identified in *R. japonica* and *R. sachalinensis* extracts, belonging to five different classes of phytochemicals (stilbenes, anthraquinones, phenylpropanoid disaccharide esters, flavan-3-ols, and procyanidins) were evaluated as potential inhibitors against SARS-CoV-2 Mpro in molecular docking study. The most successfully docked were compounds belonging to procyanidins (procyanidin B2 3,3′-di-*O*-gallate, procyanidin C1, procyanidin C1 3′,3″-di-*O*-gallate, cinnamtannin A2), phenylpropanoid disaccharide esters (vanicoside A, vanicoside B, vanicoside C, hydropiperoside, lapathoside C, tatariside B), and anthranoids (emodin, emodin bianthrone) (Table 1 and Table S1). In the case of almost all of those compounds the interactions with the catalytic residues—Cys145 and His41—were observed. Additionally, hydrogen bonds with such residues as Gly143, Ser144, Glu166, Gln189, or Thr190 were formed. Interaction with those residues were also present in case of the N3 ligand. Also, for all those compounds, except emodin bianthrone, the fitness GOLD docking scores are similar or higher than the score for N3 ligand, which could also indicate that those compounds could be classified as good inhibitors. Results from molecular docking study of phenylpropanoid disaccharide esters towards SARS-CoV-2 Mpro are presented for the first time.

Alongside these 11 compounds tested in the in vitro study, we have also tested extracts and fractions from rhizomes of *R. japonica* and *R. sachalinensis*. The results indicated that for strong inhibition of SARS-CoV-2 Mpro by *R. japonica* and *R. sachalinensis* acetone extracts (IC_50_ = 16.90 µg/mL and 9.42 µg/mL, respectively), the mainly responsible compounds are present in the butanol fractions (IC_50_ = 4.031 µg/mL for *R. sachalinensis* and IC_50_ = 7.877 µg/mL for *R. japonica*). Only these fractions revealed stronger enzyme inhibition than the corresponding acetone extracts (Table S8, Figure 6, Figure 7 and Figure 8). According to our earlier phytochemical study [33], among all obtained fractions (CH_2_Cl_2_, Et_2_O, AcOEt, *n*-BuOH, and water), the butanol fractions of *R. japonica* and *R. sachalinensis* contained the highest amount of procyanidins with high degree of polymerization such as procyanidin heptamer or octamer. Next to procyanidins, phenylpropanoid disaccharide esters were another important group of compounds detected in these fractions. The stronger inhibition of SARS-CoV-2 Mpro by *R. sachalinensis* than *R. japonica* could be associated with higher amount of procyanidins and phenylpropanoid disaccharide esters in *R. sachalinensis* rhizomes, which was confirmed in our earlier studies [32,33]. We docked compounds belonging to these phytochemical groups into the binding site of SARS-CoV-2 Mpro. Four of them: vanicoside A, B, procyanidin C1, procyanidin B2 3,3′-di-*O*-gallate, were selected to in vitro study. However, despite good results in docking study, only vanicoside A (IC_50_ = 23.10 µM) and vanicoside B (IC_50_ = 43.59 µM) showed moderate inhibition of SARS-CoV-2 Mpro. Therefore, we suggest that other compounds may be responsible for the strong inhibition of SARS-CoV-2 Mpro by butanol fractions, or a phenomenon of synergy between the compounds occurred. However, some of the well scored compounds, belonging to the phenylpropanoid esters (hydropiperoside, lapathoside C) and procyanidins (cinnamtannin A2) were not tested because of insufficient amounts obtained from the crude drug. Even so, their scores were comparable but not higher than those of the tested vanicosides/procyanidin C1, respectively. These rare compounds also occur mainly in *R.sachalinensis*, less utilized as a medicinal plant. Hence, future investigation into the anti SARS-CoV-2 potential should focus on this species.

Highly polymerized proanthocyanidins present in butanol fractions, with the degree of polymerization higher than those tested in this in vitro study (dimers, trimers), may have had a significant effect on the strong inhibitory effect of these fraction on SARS-CoV-2 Mpro. Moreover, according to previous studies [33], dimeric and trimeric proanthocyanidins (with weak inhibition activity in our experiment) and simple flavan-3-ols (epicatechin, epicatechin gallate, without inhibitory effect at 100 µM in our experiment), apart from phenylpropanoid disaccharide esters, are the main compounds in the Et_2_O and AcOEt fractions, which provides a plausible explanation of their weaker inhibitory effect (Figure 6). Additional studies are needed to confirm the inhibition of SARS-CoV-2 Mpro by highly polymerized proanthocyanidins. We are continuing isolation of these subfractions and single compounds from butanol fractions to fully confirm these assumptions. However, they are already supported by the chemical nature of these compounds as well as by the other studies outlined below. Proanthocyanidins bind proteins also non-specifically due to the numerous phenol (hydroxyl) groups that form cross-linked structures with polypeptides. This seems to depend on the size of the molecule: the more a proanthocyanidin is polymerized, the less specific the protein bonds are. Moreover, a higher degree of polymerization of proanthocyanidins increases their affinity to proteins and enhances the cross-linkages between proteins [36,37,38]. Some studies have demonstrated antiviral activity of extracts rich in proanthocyanidins. Zhuang et al. [39] found that butanol fraction of *Cinnamomi cortex* containing high amount of proanthocyanidins, inhibit SARS-CoV infection (IC_50S_ = 7.8 ± 0.3 µg/mL, wild-type SARS-CoV). Conzelmann et al. [40] revealed that black chokeberry (*Aronia melanocarpa*) juice, pomegranate (*Punica granatum*) juice and green tea (*Camellia sinensis*), rich in proanthocyanidins, possess high antiviral efficacy against SARS-CoV-2 (BetaCoV/France/IDF0372/2020) and influenza A virus (A/H1N1/Brisbane/59/2007). The 5-min incubation of SARS-CoV-2 with black chokeberry juice resulted in a ≥ 1.52 log_10_ decrease in infectivity (which corresponds to a 96.98% reduction of infectivity). The mean procyanidin polymerization degree (mDP) in *A. melanocarpa* juice is high: 12–52 [41]. Due to general affinity of proanthocyanidins to proteins, in addition to binding the main protease SARS-CoV-2, they can also bind other essential viral structural proteins. So far, an inhibitory effect of extracts rich in proanthocyanidins or isolated compounds on the other enveloped viruses such as influenza and RSV has been observed [42,43,44]. It was shown that the *Rumex acetosa* extract rich in proanthocyanidins and its main active constituent-procyanidin B2-di-gallate protect cells from influenza A virus infection by inhibiting viral attachment [44]. Similarly, in the case of the herpes simplex virus (HSV-1), inhibition of virus adsorption and penetration by proanthocyanidins was observed [45]. Moreover, proanthocyanidins were proposed as a new class of hepatitis B and D virus entry inhibitors [46]. They directly target the preS1 region of the HBV large surface protein.

Taking into account the above properties of proanthocyanidins, in the context of COVID-19, it has been hypothesized that proanthocyanidins can inhibit the attachment of SARS-CoV-2 to the oral epithelium [47] and lower viral adsorption and penetration. The lower viral load may lower the risk of developing severe condition [48]. It was suggested that pharmaceutical preparations like gargle and mouthwash solutions as well as lozenges or chewing gums with extracts rich in proanthocyanidins may be useful in the prophylaxis and adjunctive therapy of COVID-19. An important issue in the case SARS-CoV-2 treatment, apart from virus neutralization, is also dealing with overreaction of the immune system and a cytokine storm leading to systemic inflammatory response syndrome (SIRS) and ARDS [48]. Importantly, phytochemicals, including proanthocyanidins, present in the studied extracts and fractions have proven anti-inflammatory effects [49]. However, this issue goes beyond the scope of the present work and requires a separate development.

## 4. Materials and Methods

### 4.1. Extracts and Fractions of Reynoutria Species

The plant material, extracts, and fractions were obtained according to procedures described in our previous paper [33], stored under −80 °C and their composition confirmed to be unchanged until the beginning of current experiments.

### 4.2. Compounds

Vanicoside A and vanicoside B were isolated earlier from rhizomes of *Reynoutria sachalinensis* (F.Schmidt) Nakai, according procedure described in [32]. The structures of vanicoside B and vanicoside A were identified using 1H and 13C NMR and HR-MS-qTOF MS analysis and presented in the above article [32]. Emodin, piceid, epigallocatechin gallate, procyanidin B2, procyanidin C1, were purchased in ChemFaces (Wuhan, China), procyanidin B2 3,3′-di-*O*-gallate was purchased in Albtechnology (HongKong) and resveratrol, epicatechin, epicatechin gallate in MilliporeSigma (St. Louis, MO, USA) and their purity verified using HPLC and exceeded 98%.

### 4.3. Molecular Docking

The 25 selected compounds were docked into the binding site of SARS-CoV-2 main protease. Molecular docking was carried out with GOLD software (version 5.7.2), which uses genetic algorithms for generation of ligand conformations [50]. Crystal structure of main SARS-CoV-2protease was obtained from PDB database [51] (PDB code: 6LU7). Binding site was defined based on the position of co-crystalized ligand (N3) from PDB protein structure and all atoms within 10 Å from the ligand were selected. First, the N3 was re-docked into the binding site of the main protease and RMSD with respect to co-crystalized ligand was calculated. Interactions of best pose of re-docked ligand were compared to the interactions of N3 ligand provided in the literature such as: Gly143, Cys145, His163, His164, Glu166, Gln189, Thr190 [52]. Docking protocol, where ligands were flexible, and residues of the binding site were set to rigid, was established and selected compounds were docked into the binding site of the receptor. Even though most of the ligands were large and flexible molecules, none of the rotatable bonds of ligands were fixed and default setting or ligand flexibility were used. Genetic algorithm used in GOLD is able to predict the binding mode of highly flexible molecules [50]. Structures of ligands were obtained from PubChem database in sdf format [53]. In case of lack of 3D structure, 2D structures were uploaded to docking software. Interaction analyses of protein-ligand complexes and 2D interaction diagrams were done with BIOVIA Discovery Studio 2020 [54]. The 3D interaction visualizations of ligands with Mpro were created with PYMOL version 2.3.5 [55]. GoldScore scoring function was chosen for ranking of compounds and for each ligand 10 poses were generated.

Molecular docking analyses and choice of the best candidates for Mpro inhibitors was based on visual inspection of protein-ligand complexes. When interactions with key residues were observed—those compounds were suggested as potential candidates for inhibitors. Key residues were defined based on the literature [52] and by re-docking the co-crystalized ligand. Additionally, GOLD fitness scores of all docked compounds were analyzed.

### 4.4. Inhibition of SARS-CoV-2 M^pro^ Enzyme-In Vitro Study

The experiment was performed according to the procedure described in the previous article [56] with minor modifications. All experiments were carried out in 96-well assay plates. Buffer solution (pH 7.3) contained 50 mM Tris, 1 mM EDTA, and 1 mM DTT. Inhibitor screenings: to the wells, 1 µL of DMSO inhibitors solutions was added. Then, 79 µL of SARS-CoV-2 Mpro enzyme (E) in buffer was added. Enzyme was incubated with inhibitors (I) for 10′ at 37 °C. After incubation, 20 µL of substrate (QS1, Ac-Abu-Tle-Leu-Gln-ACC) [15] in buffer solution was added. Final concentrations were [E] = 100 nM, [QS1] = 50 µM, [I] = 100 µM for compounds 1–11 or [I] = 50 µg/mL for extracts and fractions 12–23. Measurements were carried out in Molecular Devices SpectraMax Gemini XPS spectrofluorometer at 37 °C for 30′. Liberation of ACC fluorophore was measured using λ_ex_ = 355 nm and λ_em_ = 460 nm wavelengths. The linear range of progress curves was used for analysis. Measurements were carried out at least in triplicate. The results were presented as mean values with standard deviations. Compounds as well as extracts and fractions displaying >20% inhibition during the screening were selected for further analysis.

Determination of enzyme inhibition % in serial dilutions of inhibitors: serial dilutions in DMSO of selected compounds were prepared (dilution factor 2/3). The experiment was carried out analogically to the screening described above. 1 µL of diluted inhibitors in DMSO were added to the wells. Then, 79 µL of SARS-CoV-2 Mpro enzyme in buffer was added. Enzyme was incubated with inhibitors for 10′ at 37 °C. After incubation, 20 µL of substrate (QS1) in buffer solution was added. Final concentrations were [E] = 100 nM and [QS1] = 50 µM. Measurements were carried out in Molecular Devices SpectraMax Gemini XPS spectrofluorometer at 37 °C for 30′. Liberation of ACC fluorophore was measured using λ_ex_ = 355 nm and λ_em_ = 460 nm wavelengths. The linear range of progress curves was used for analysis. Measurements were carried out at least in triplicate. The results were presented as mean values with standard deviations.

### 4.5. Statistical Analysis

Each assay was performed in at least triplicate and presented as mean ± SD for *n* ≥ 3. Statistical analysis was performed using GraphPad Prism v.7 software (GraphPad Software, San Diego, CA, USA). Initially, the Shapiro-Wilk test was used to assess the distribution of results. Then, in the Student t-test a comparison of the means between the treated and control samples (DMSO instead of the inhibitor) was used. Results with *p* ≤ 0.05 was considered statistically significant. Log IC_50_, IC_50_ and R^2^ were calculated using GraphPad Prism v. 7.

## 5. Conclusions

Among the 11 phytochemicals (vanicoside A, vanicoside B, resveratrol, piceid, emodin, epicatechin, epicatechin gallate, epigallocatechin gallate, procyanidin B2, procyanidin C1, procyanidin B2 3,3’-di-*O*-gallate) selected for in vitro study after docking into the binding site of SARS-CoV-2 Mpro, the best results were achieved with vanicoside A and vanicoside B with moderate inhibition of SARS-CoV-2 Mpro, equal IC_50_ = 23.10 µM and 43.59 µM, respectively. These compounds are important components of the extracts and fractions obtained from the rhizomes of *Reynoutria japonica* and *Reynoutria sachalinensis*. As the first report about an interaction of these compounds with a virus protein, it also indicates a possible biological function in the plant pathogen resistance that should inspire further studies.

Nonetheless, the evident inhibitory activity of the vanicosides does not alone explain a strong inhibition of SARS-CoV-2 Mpro by acetone extracts and mainly butanol fractions. As the main constituents of butanol fractions, besides the phenylpropanoid disaccharide esters (e.g., vanicosides), are highly polymerized procyanidins, we suppose that they could be responsible for strong inhibitory properties of these fractions. Moreover, the less polymerized procyanidins (procyanidin B2, procyanidin C1, procyanidin B2 3,3’-di-*O*-gallate), abundantly present in the fractions with weaker inhibition of the enzyme, showed no remarkable activity. Further studies are needed to prove the contribution of highly polymerized procyanidins and their potential synergy with vanicosides.

## Figures and Tables

**Table 1:** 2D interactions diagrams of presumably good candidates for Mpro inhibitors.

Phenylpropanoid Disaccharide Esters	
Vanicoside B 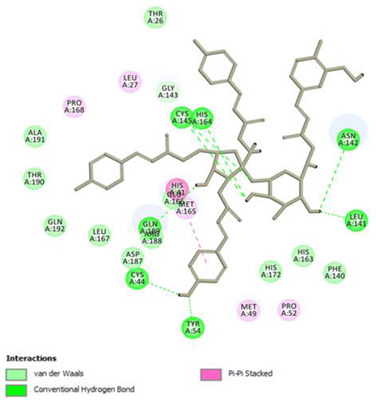 Conventional Hydrogen Bond: **Cys145**, **Gln189**, His164, Asn142, Leu141, Tyr54, Cys44; Pi-interactions: **His41**, Met165	Vanicoside A 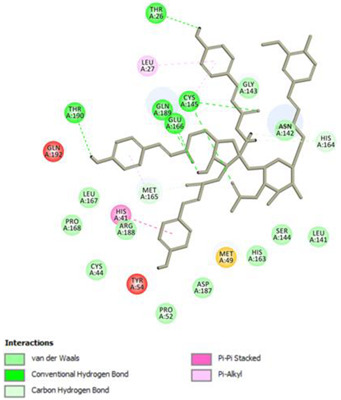 Conventional Hydrogen Bond: **Cys145**, **Glu166**, **Gln189**, **Thr190**, Thr26; Pi-interactions: **His41**, Leu27
**Procyanidins**	
Procyanidin C1 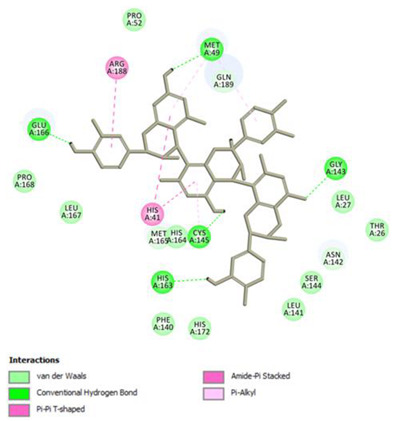 Conventional Hydrogen Bond: **Cys145**, Met49, **Glu166**, **Gly143**, His163; Pi-interactions: **His41**, Arg188, Met49	Procyanidin B2 3,3’-di-*O*-gallate 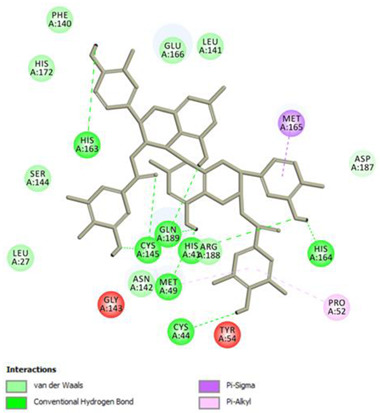 Conventional Hydrogen Bond: **His41**, **Cys145**, His163, His164, Cys44, Met49, **Gln189**; Pi-interactions: Pro52, Met165
**Anthraquinone**	
Emodin 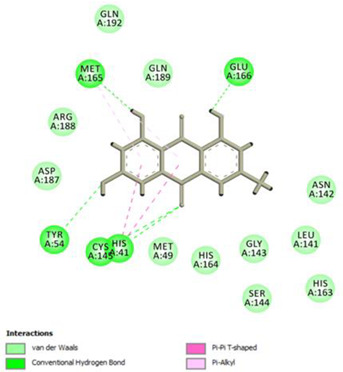 Conventional Hydrogen Bond: **Cys145**, **Glu166**, Met165, Tyr54, His41; Pi-interactions: **His41**; Met165	

**Table 2:** 2D interactions diagrams of presumably not good candidates for Mpro inhibitors.

Stilbenes	
**Resveratrol** 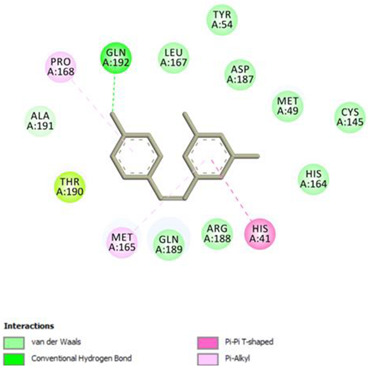 Conventional Hydrogen Bond: Gln 192; Pi-interactions: **His41**, Pro168, Met165	Piceid 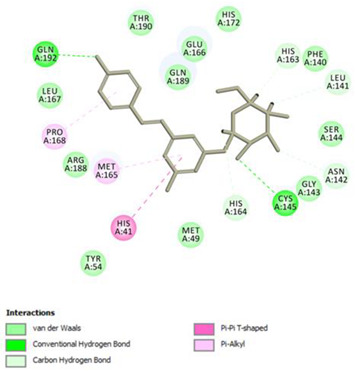 Conventional Hydrogen Bond: **Cys145**, Gln192; Pi-interactions: **His41**, Met165, Pro168
**Flavanols and Procyanidins**	
**(−)-Epigallocatechin gallate** 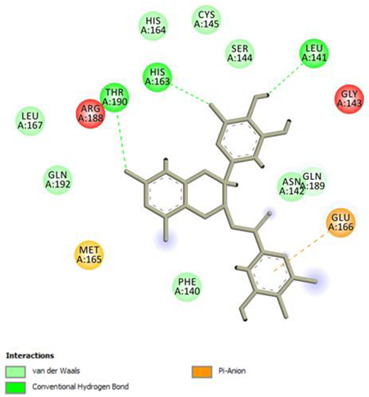 Conventional Hydrogen Bond: **Thr190**, Leu141, His163; Pi-interactions: Glu166	Epicatechin 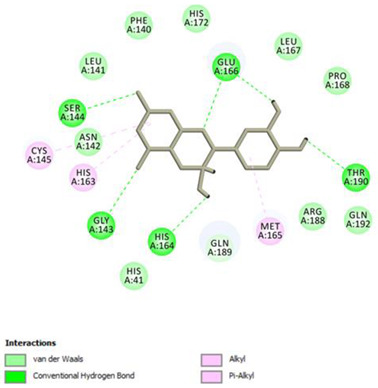 Conventional Hydrogen Bond: **Ser144**, **Glu166**, **Gly143**, His164, **Thr190**; Pi-interactions: Met165, His163, Cys145
Epicatechin gallate 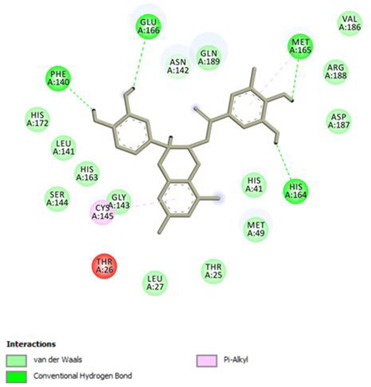 Conventional Hydrogen Bond: Met165, His164, **Glu166**, Phe140; Pi-interactions: Cys145, Met165	Procyanidin B2 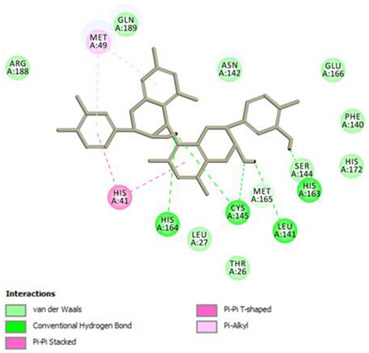 Conventional Hydrogen Bond: **Cys145**, Leu141, His163, His164; Pi-interactions: **His41**, Met49

## Data Availability

Data is contained within the article and supplementary materials.

## Supplementary Materials

The following are available online at https://www.mdpi.com/article/10.3390/ph14080742/s1, Figure S1: Structures of compounds docked into the binding site of SARS-CoV-2 main protease, generated with PubChem Sketcher V2.4 (PubChem Sketcher V2.4), Table S1: Compounds docked to SARS-CoV-2 main protease of (Mpro), Table S2: GOLD docking scores of all compounds tested in vitro, Table S3: Compounds studied in vitro against the proteases SARS-CoV-2 Mpro, Table S4: Extracts and fractions studied in vitro against the proteases SARS-CoV-2 Mpro, Table S5: SARS-CoV-2 Mpro activity in serial dilution of isolated compounds. The results were presented as SARS-CoV-2 Mpro residual activity (%), Table S6: SARS-CoV-2 Mpro activity in serial dilution of extracts and fractions from *R. japonica* (R.j.) and *R. sachalinensis* (R.s.) rhizomes. The results were presented as SARS-CoV-2 Mpro residual activity (%), Table S7: SARS-CoV-2 Mpro activity in serial dilution of isolated compounds. The results were presented as SARS-CoV-2 Mpro inhibition (%), Table S8: SARS-CoV-2 Mpro activity in serial dilution of extracts and fractions from *R. japonica* (R.j.) and *R. sachalinensis* (R.s.) rhizomes. The results were presented as SARS-CoV-2 Mpro inhibition (%).
