# Supplementary material for: Reynoutria Rhizomes as a Natural Source of SARS-CoV-2 Mpro Inhibitors–Molecular Docking and In Vitro Study
Source: Pharmaceuticals (Basel). 2021 Jul 29;14(8):742. doi: 10.3390/ph14080742 (PMC8399519; doi:10.3390/ph14080742)
Supplement: Supplementary file 1 [file pharmaceuticals-14-00742-s001.zip › pharmaceuticals-1289886-SI.pdf]

SUPPLEMENTARY MATERIAL

Reynoutria rhizomes as a natural source of SARS-CoV-2 Mpro inhibitors - molecular docking and *in vitro* study.

Izabela Nawrot-Hadzik, Mikołaj Żmudziński, Adam Matkowski, Robert Preissner, Małgorzata Kęsik-Brodacka, Jakub Hadzik, Marcin Drąg, Renata Abel

resveratroloside
CID: 71528811

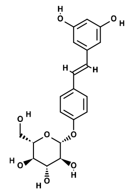

emodin
CID: 3220

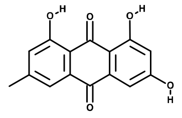

vanicoside B
CID: 10033855

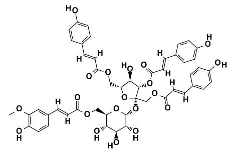

tatariside B
CID: 102450498

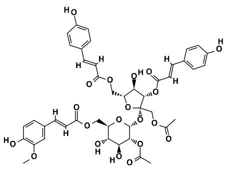

procyanidin B2 3'-O-gallate
CID:15593124

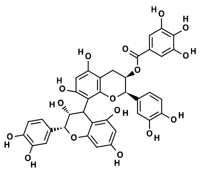

piceatannol
CID: 667639

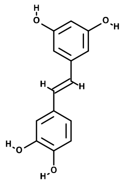

emodin 8-glucoside
CID: 99649

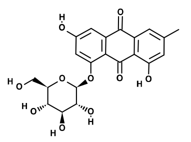

vanicoside C
CID: 10724147

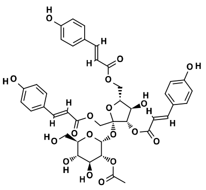

Epicatechin
CID: 72276

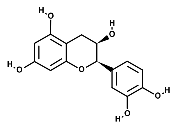

procyanidin B2 3,3'-di-O-gallate
CID: 124016

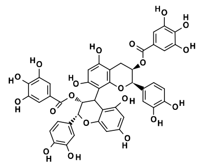

piceatannol glucoside
CID:131752959

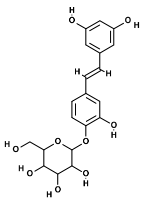

emodin bianthrone
CID: 10097848

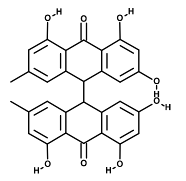

hydropiperoside
CID: 10350284

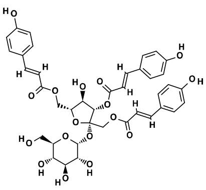

epicatechin gallate
CID: 107905

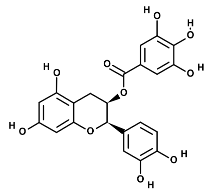

procyanidin C1
CID: 169853

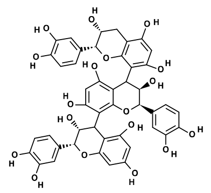

piceid
CID: 5281718

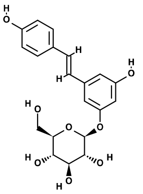

physcion
CID: 10639

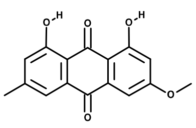

lapathoside A
CID: 10011201

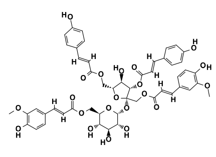

epigallocatechin gallate
CID: 65064

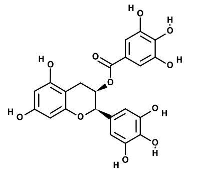

procyanidin C1 3',3''-di-O-gallate
CID: 73822578

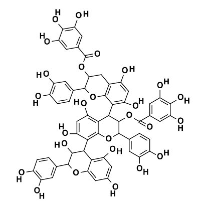

resveratrol
CID: 445154

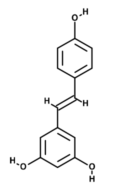

vanicoside A
CID: 6449878

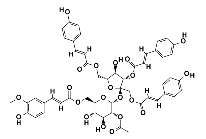

lapathoside C
CID: 11061786

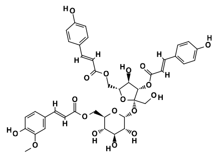

procyanidin B2
CID: 122738

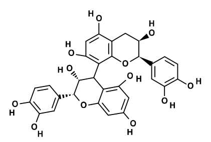

cinnamtannin A2
CID: 16130899

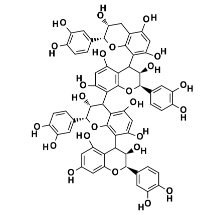

Supplementary Figure S1. Structures of compounds docked into the binding site of SARS-CoV-2 main protease, generated with PubChem Sketcher V2.4 (PubChem Sketcher V2.4).

Supplementary Table S1. Compounds docked to SARS-CoV-2 main protease of (Mpro).

	Compound name	2D interaction diagram
***Reynoutria japonica* I stilbenes**		
1	**Resveratroloside**	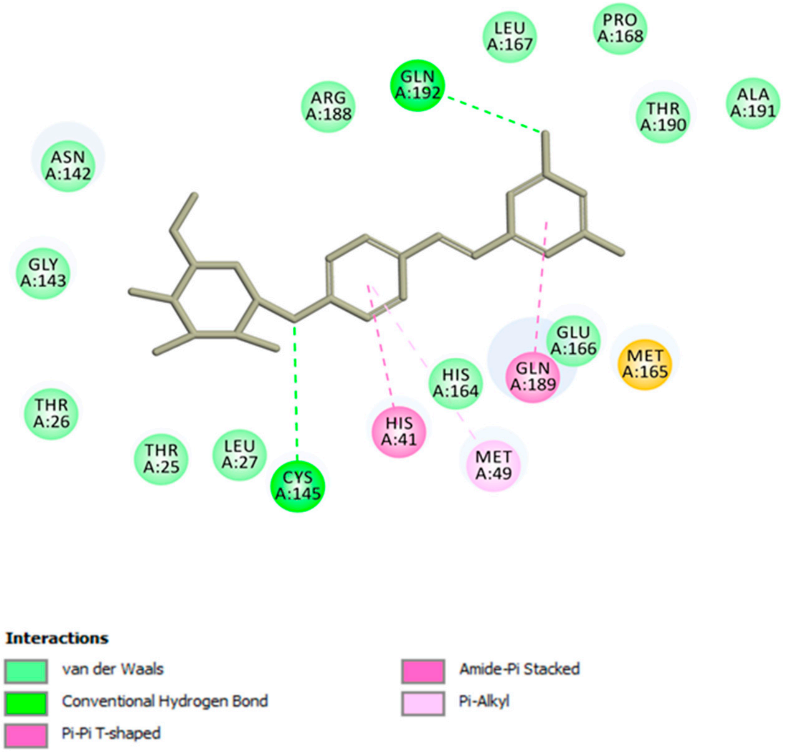
2	**Piceatannol glucoside**	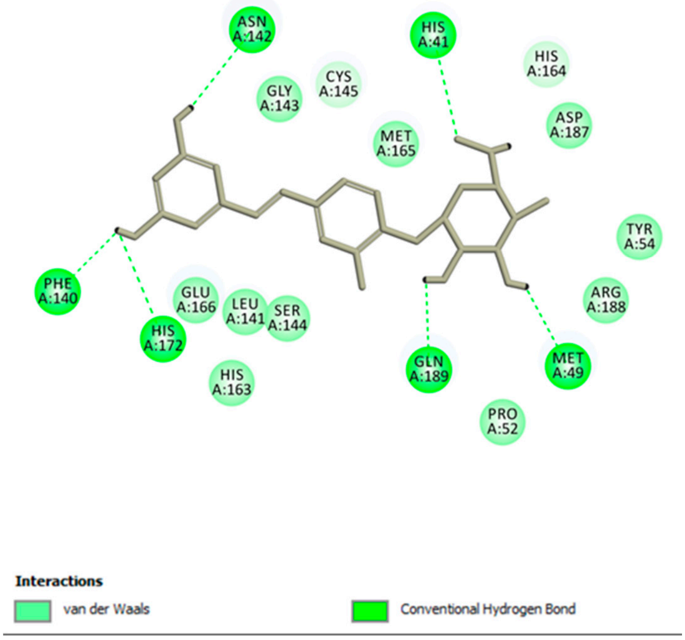

<table>
<tr><td>3</td><td>Piceatannol</td><td>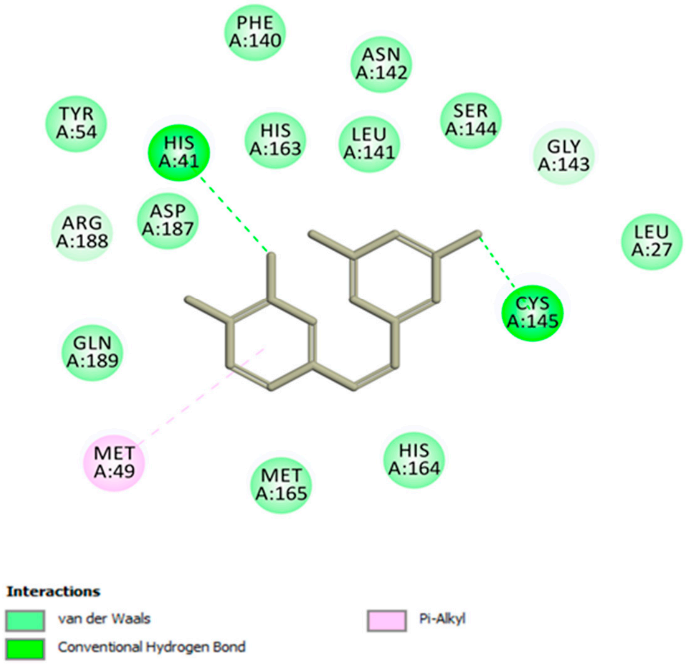
</td></tr>
<tr><td colspan="3">R.japonica and R.sachalinensis | procyanidins</td></tr>
<tr><td>4</td><td>Procyanidin B2 3'-O-gallate</td><td>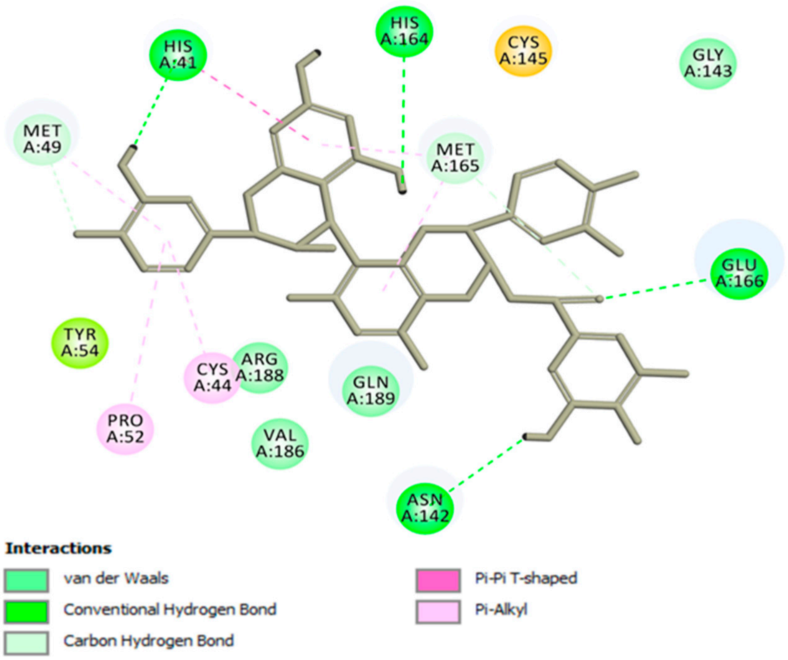
</td></tr>
</table>

5	Procyanidin C1 3',3''-di-O-gallate	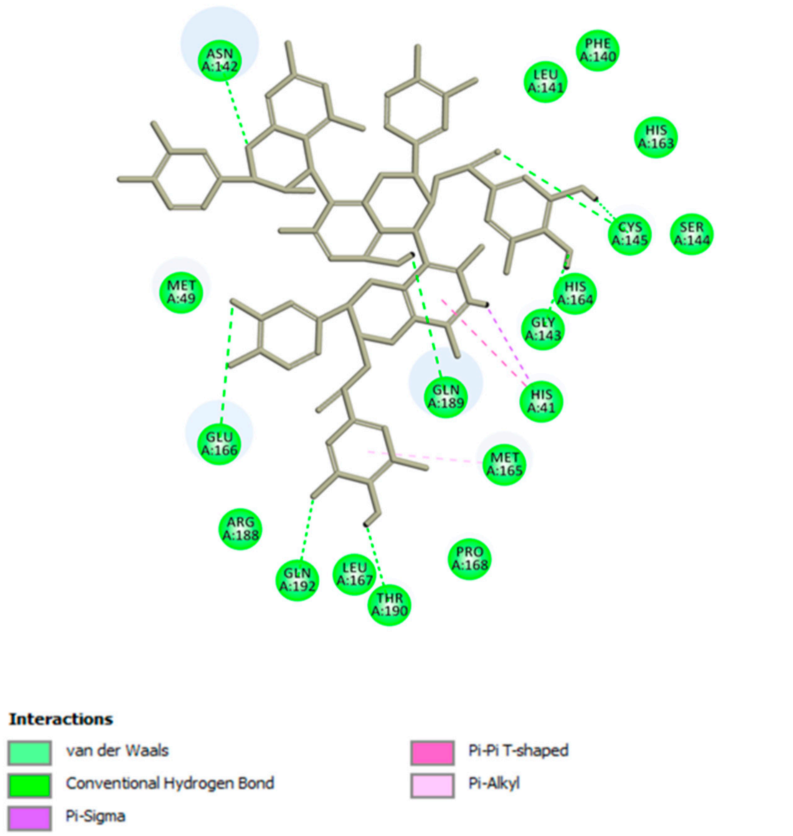
6	Cinnamtannin A2	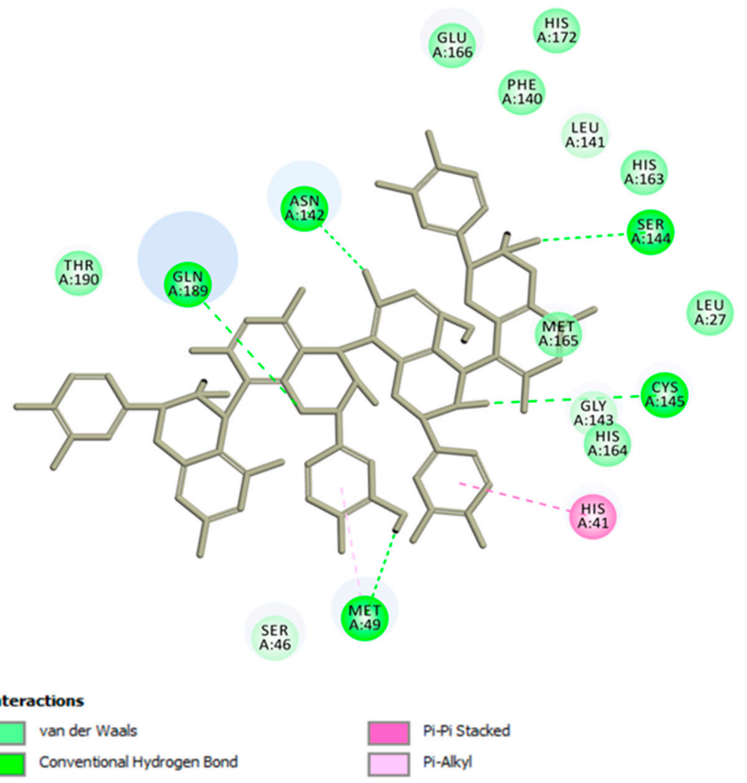

7	**Physcion**	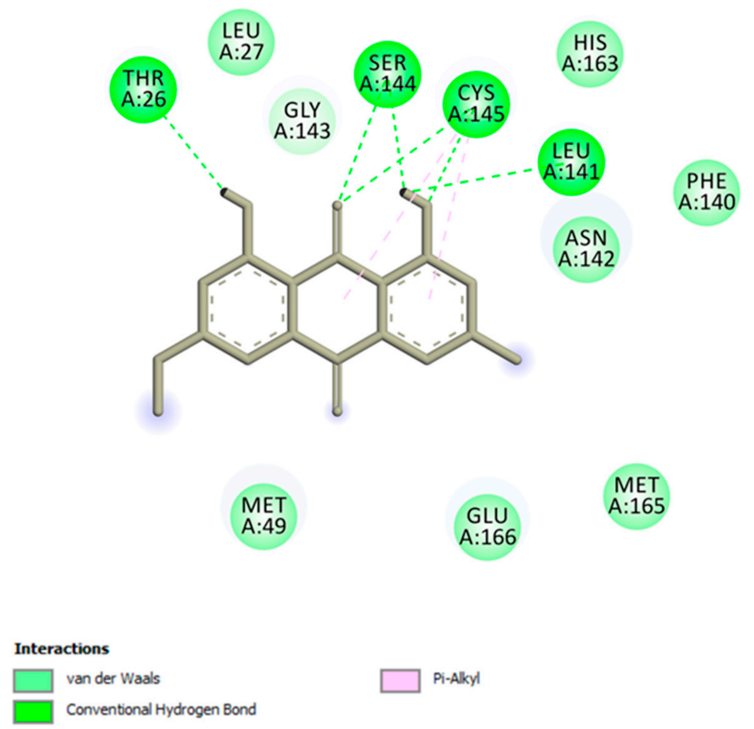
8	**Emodin bianthrone**	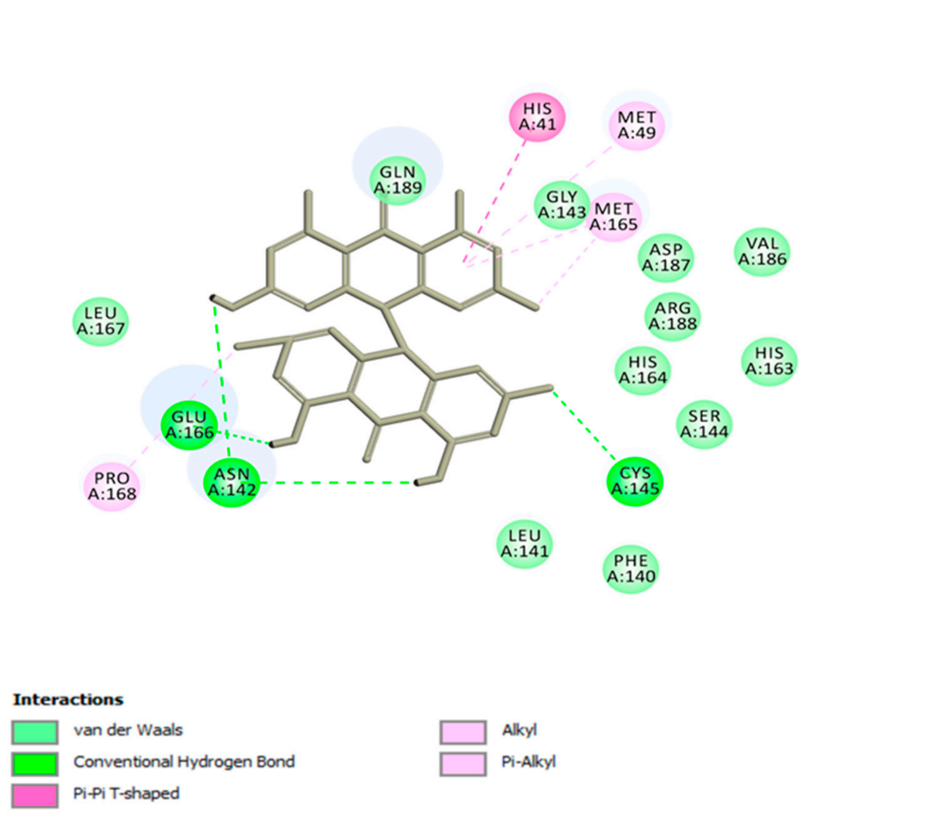

<table>
<tr><td>9</td><td>Emodin-8-glucoside</td><td>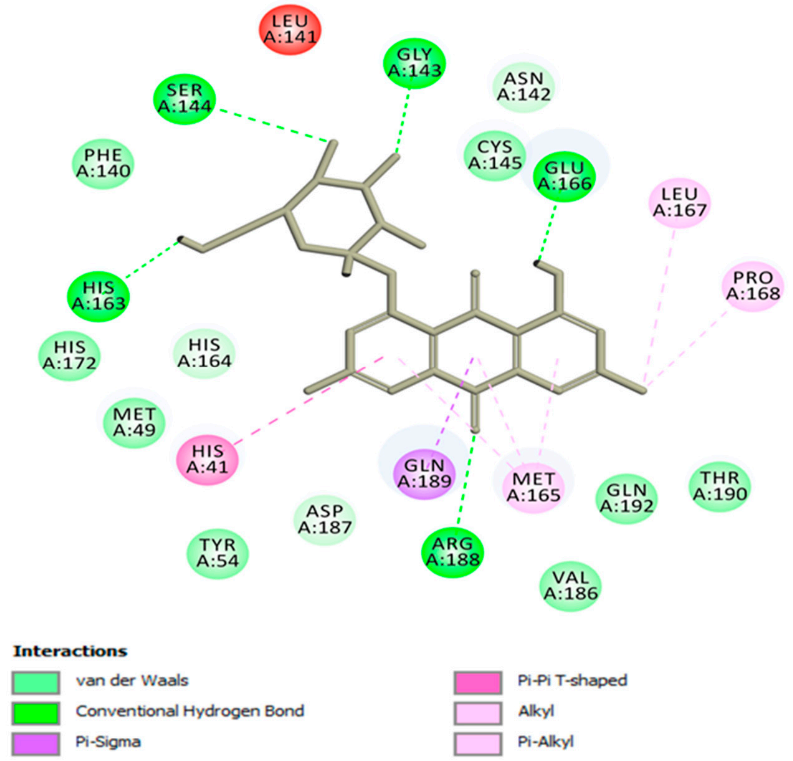
</td></tr>
<tr><td colspan="3">Mainly R. sachalinensis | Phenylpropanoid Disaccharide Esters</td></tr>
<tr><td>10</td><td>Hydropiperoside</td><td>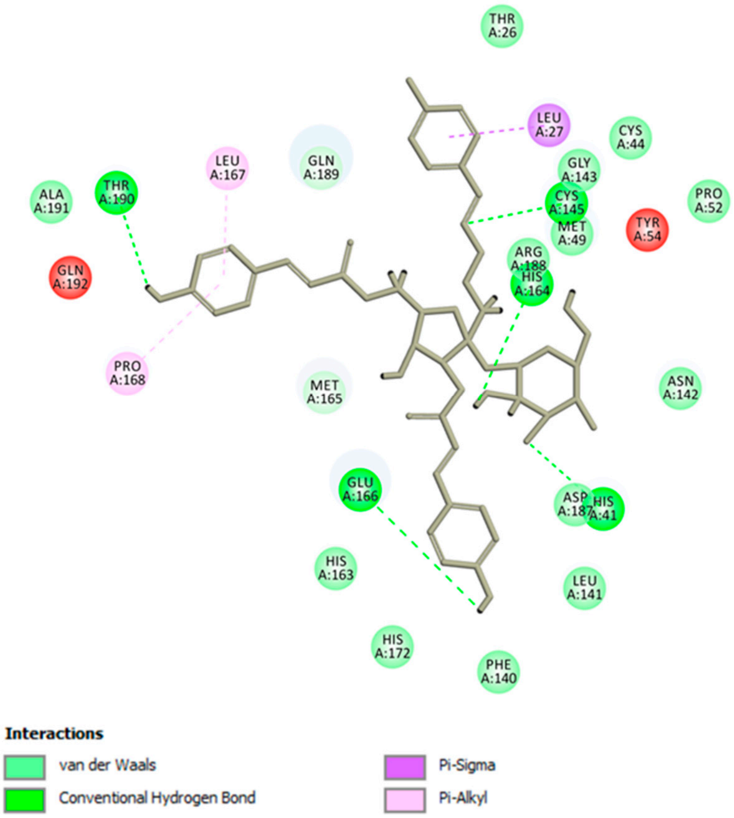
</td></tr>
</table>

11	Lapathoside A	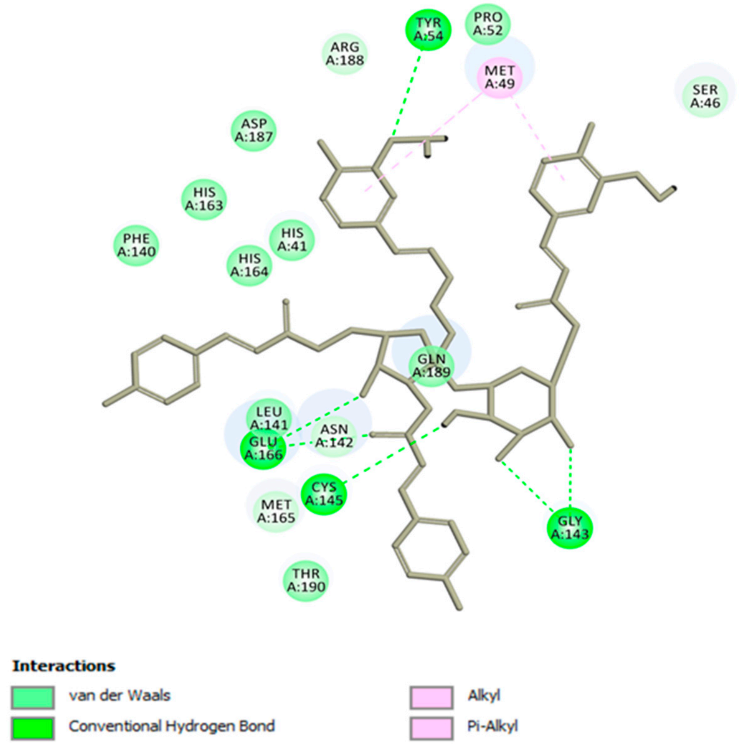
12	Tatariside B	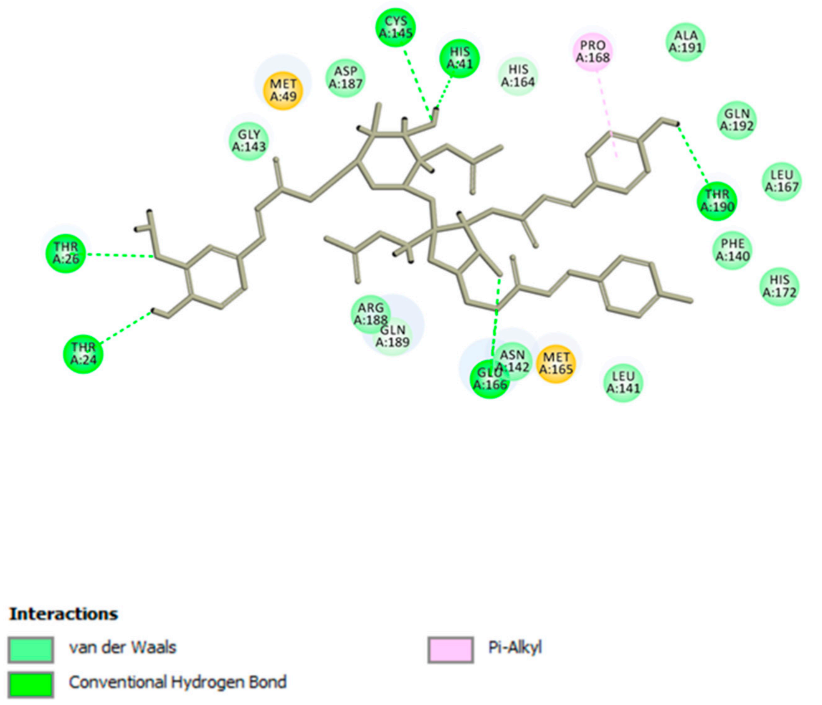

13	Vanicoside C	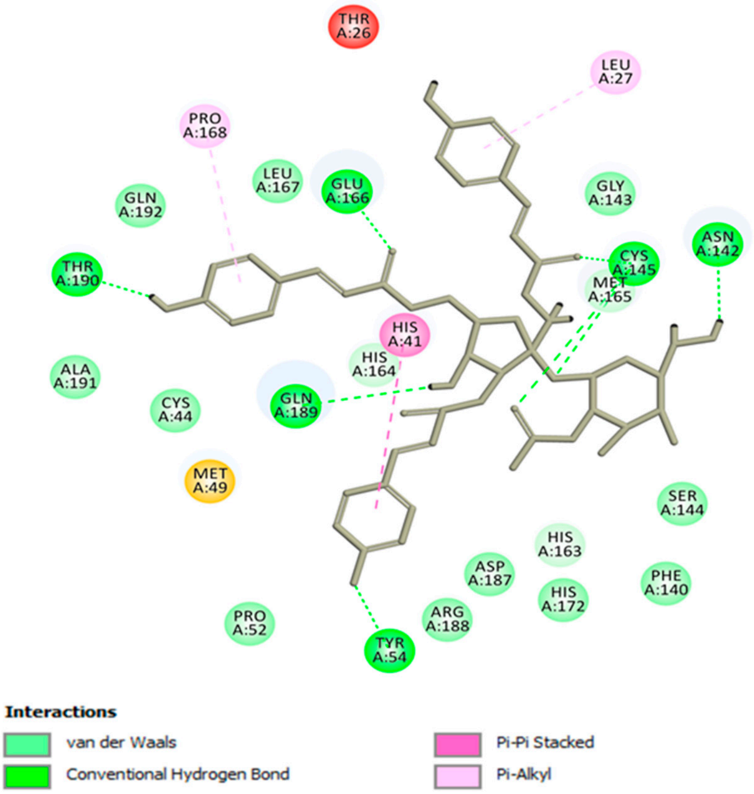
14	Lapathoside C	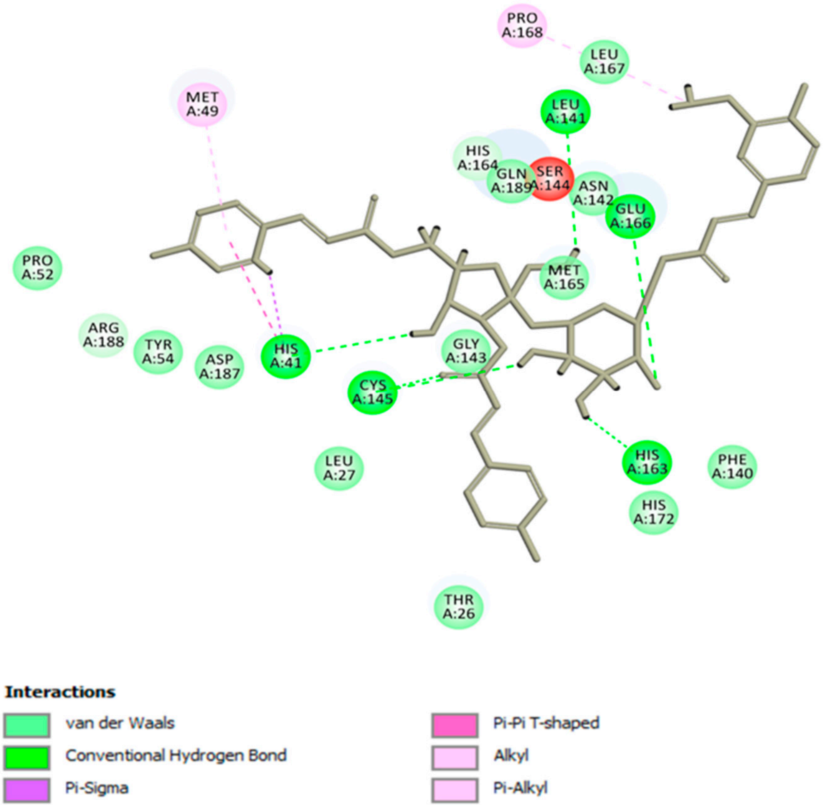

Supplementary Table S2. GOLD docking scores of all compounds tested in vitro.

Ligand name	Goldscore.Fitness	**Goldscore.External.HBond**	**Goldscore.External.Vdw**	**Goldscore.Internal.HBond**	**Goldscore.Internal.Torsion**	**Goldscore.Internal.Vdw**
**N3**	86.5557	4.7025	62.1031	0.0000	-36.2638	-337.6012
			in vitro tested			
**Vanicoside A**	**115.7695**	0.7297	84.8721	0.0000	-95.3087	-2322.0438
**Vanicoside B**	**129.6954**	3.4584	92.7738	0.0000	-89.6407	-2184.0755
**Emodin**	**92.9682**	2.0399	69.8483	0.0000	-92.4773	-2324.0449
**Procyanidin B2 3,3'-di-*O*-gallate**	**99.5746**	1.0515	83.7998	0.0000	-48.7760	-1436.3108
**Procyanidin C1**	**103.3066**	2.6481	78.1660	0.0000	-26.5339	-1583.1373
Resveratrol	48.7919	5.5779	31.6672	0.0000	-5.9684	-4.2557
Piceid	68.7376	5.1522	46.8220	0.0000	-9.8268	-6.9843
(-)-Epigallocatechin gallate	71.1295	5.5336	49.3404	0.0000	-10.0207	-7.3450
Epicatechin	69.4952	3.2339	50.2172	0.0000	-7.6040	-6.6429
Epicatechin gallate	56.0065	3.9760	39.8699	0.0000	-57.7864	-2294.9008
Procyanidin B2	85.8317	3.2692	65.2979	0.0000	-19.4849	-1005.2852
			not tested *in vitro*			
Resveratroloside	52.7316	0.0000	39.7201	0.0000	-11.6108	-4.2389
Piceatannol glucoside	50.8886	0.8703	37.1399	0.0000	-12.8567	-5.1795
Piceatannol	40.3628	6.0000	25.6313	0.0000	-0.2520	-0.1850
Procyanidin B2 3'-*O*-gallate	78.3216	0.3099	67.9252	0.0000	-32.7348	-1161.8064
Procyanidin C1 3',3''-di-*O*-gallate	82.6413	1.6844	72.6499	0.0000	-59.0013	-2328.1308
**Cinnamtannin A2**	**103.2133**	0.5067	78.6915	0.0000	-36.7637	-2137.3809
Physcion	70.1055	4.0930	51.3237	0.0000	-9.1274	-6.5047
Emodin bianthrone	54.8997	0.1714	42.4375	0.0000	-11.1523	-0.1254
Emodin-8-glucoside	69.4952	3.2339	50.2172	0.0000	-7.6040	-6.6429
**Hydropiperoside**	**111.3499**	2.6981	91.0385	0.0000	-72.5777	-1814.3937
Lapathoside A	75.8767	0.5704	63.9491	0.0000	-90.7232	-2352.9685
Tatariside B	86.0933	3.6217	61.5218	0.0000	-84.1370	-2293.6888
Vanicoside C	95.2201	1.6549	94.6805	0.0000	-72.4706	-1985.4662
**Lapathoside C**	**105.5444**	2.4506	76.7571	0.0000	-76.2612	-1989.8717

Supplementary Table S3. Compounds studied in vitro against the proteases SARS-CoV-2 Mpro.

**No.**	**Compound**	**Molecular weight g/mol**	**Inhibitor concentration μM**	**Amount used in the test* μg**	**SARS-CoV-2 Mpro residual activity [% ± S.D.]**
1	Vanicoside A	998.9	100	9.99	**29.3 ± 2.9**
2	Vanicoside B	956.9	100	9.57	**36.2 ± 2.2**
3	Resveratrol	228.24	100	2.28	**87.5 ± 0.8**
4	Piceid	390.4	100	3.90	**91.6 ± 0.5**
5	Emodin	270.24	100	2.70	**48.5 ± 0.5**
6	Epicatechin	290.27	100	2.90	**103.0 ± 3.3**
7	Epicatechin gallate	442.4	100	4.42	**100.7 ± 3.8**
8	Epigallocatechin gallate	458.4	100	4.58	**89.3 ± 4.6**
9	Procyanidin B2	578.5	100	5.79	**86.1 ± 1.6**
10	Procyanidin C1	866.8	100	8.67	**77.7 ± 7.4**
11	Procyanidin B2 3,3'-di-O-gallate	882.7	100	8.83	**63.3 ± 3.2**

* the amount of the compound added to the reaction mixture; for easier comparison with the extracts and fractions. S.D – standard deviation

Supplementary Table S4. Extracts and fractions studied in vitro against the proteases SARS-CoV-2 Mpro.

No.	Extract/fraction	Inhibitor concentration µg/ml	Amount used in test* µg	SARS-CoV-2 Mpro residual activity [% ± S.D.]
12	*R. japonica* acetone extract	50	5.0	**16.4 ± 1.4**
13	*R. sachalinensis* acetone extract	50	5.0	**6.6 ± 0.6**
14	*R. japonica* dichloromethane	50	5.0	**34.5 ± 1.2**
15	*R.sachalinensis* dichloromethane	50	5.0	**21.6 ± 0.6**
16	*R. japonica* diethyl ether	50	5.0	**49.0 ± 1.1**
17	*R. sachalinensis* diethyl ether	50	5.0	**47.6 ± 0.6**
18	*R. japonica* ethyl acetate	50	5.0	**20.9 ± 0.4**
19	*R. sachalinensis* ethyl acetate	50	5.0	**38.8 ± 1.9**
20	*R. japonica* butanol	50	5.0	**9.0 ± 0.6**
21	*R. sachalinensis* butanol	50	5.0	**2.6 ± 0.1**
22	*R. japonica* water	50	5.0	**38.1 ± 1.6**
23	*R. sachalinensis* water	50	5.0	**10.7 ± 0.6**

Supplementary Table S5. SARS-CoV-2 Mpro activity in serial dilution of isolated compounds. The results were presented as SARS-CoV-2 Mpro residual activity [%].

	**1**	**2**	**5**	**10**	**11**
Concentr. [μM]	Vanicoside A	Vanicoside B	Emodin	Procyanidin C1	Procyanidin B2 3,3'-di-O-gallate
100	29.3 ± 2.9	36.2 ± 2.2	**48.5 ± 0.5**	77.7 ± 7.4	63.3 ± 3.2
66.7	35.2 ± 5.4	**51.8 ± 1.5**	72.5 ± 1.6	131.3 ± 7.9	99.0 ± 4.3
44.4	42.3 ± 5.8	65.9 ± 1.6	80.5 ± 2.6	147.4 ± 3.7	130.7 ± 7.3
29.6	**52.3 ± 6.4**	70.8 ± 7.4	87.5 ± 4.1	149.6 ± 7.4	148.1 ± 4.2
19.8	73.7 ± 5.7	97.8 ± 2.5	99.9 ± 2.3	169.1 ± 2.3	156.9 ± 6.7
13.2	91.8 ± 2.7	111.8 ± 7.9	111.0 ± 6.4	177.3 ± 8.3	164.9 ± 4.1
8.78	107.0 ± 1.2	125.8 ± 1.7	118.6 ± 4.5	175.9 ± 5.1	162.9 ± 5.9
5.85	111.1 ± 6.2	133.4 ± 2.4	132.0 ± 3.2	175.1 ± 6.5	164.7 ± 5.2
3.90	123.9 ± 3.2	138.5 ± 5.4	133.7 ± 4.2	171.0 ± 8.3	167.5 ± 3.8

Supplementary Table S6. SARS-CoV-2 Mpro activity in serial dilution of extracts and fractions from R. japonica (R.j.) and R.sachalinensis (R.s.) rhizomes. The results were presented as SARS-CoV-2 Mpro residual activity [%].

	**12**	**13**	**14**	**15**	**16**	**17**	**18**	**19**	**20**	**21**	**22**	**23**
Concentr. [μg/ml]	*R.j.* acetone	*R.s.* acetone	*R.j.* DCM	*R.s.* DCM	*R.j.* DEE	*R.s.* DEE	*R.j.* EA	*R.s.* EA	*R.j.* butanol	*R.s.* butanol	*R.j.* water	*R.s.* water
50.0	16.4 ± 1.4	6.6 ± 0.6	34.5 ± 1.2	21.6 ± 0.6	49.0 ± 1.1	47.6 ± 0.6	20.9 ± 0.4	38.8 ± 1.9	9.0 ± 0.6	2.6 ± 0.1	38.1 ± 1.6	10.7 ± 0.6
33.3	26.5 ± 2.5	8.4 ± 2.3	56.9 ± 2.1	41.0 ± 1.4	83.4 ± 4.3	91.1 ± 7.7	49.0 ± 2.6	43.2 ± 1.7	12.5 ± 0.5	6.4 ± 0.2	53.9 ± 1.2	18.4 ± 0.9
22.2	41.5 ± 2.8	14.8 ± 2.3	70.2 ± 4.6	55.0 ± 0.8	105.5 ± 2.1	120.1 ± 3.1	81.0 ± 0.5	68.9 ± 3.3	20.8 ± 1.4	9.4 ± 0.8	84.4 ± 1.3	33.7 ± 0.9
14.8	65.8 ± 3.0	28.8 ± 2.8	88.5 ± 5.9	67.3 ± 1.7	132.5 ± 6.5	148.4 ± 2.9	102.6 ± 6.3	95.5 ± 2.0	33.6 ± 1.9	19.0 ± 1.1	111.7 ± 2.3	51.5 ± 1.8
9.88	87.1 ± 3.2	49.4 ± 2.5	104.3 ± 1.5	95.0 ± 1.5	144.6 ± 9.6	168.2 ± 4.0	130.5 ± 7.1	130.9 ± 8.1	43.0 ± 0.7	31.6 ± 2.0	131.3 ± 0.6	66.3 ± 0.8
6.58	105.4 ± 4.2	78.3 ± 3.7	122.4 ± 1.8	128.9 ± 5.5	161.3 ± 3.9	179.7 ± 6.2	146.8 ± 2.2	143.2 ± 6.2	63.4 ± 1.3	42.9 ± 2.6	143.4 ± 2.8	95.2 ± 5.1
4.39	117.1 ± 7.2	98.4 ± 0.7	135.0 ± 3.3	154.4 ± 2.0	171.0 ± 6.7	187.2 ± 4.1	158.8 ± 7.0	178.7 ± 6.8	91.4 ± 8.3	60.8 ± 5.8	164.4 ± 8.8	121.3 ± 4.0
2.93	123.3 ± 5.7	116.9 ± 1.7	137.0 ± 9.8	174.5 ± 2.1	178.5 ± 2.8	189.5 ± 1.1	168.1 ± 3.7	170.6 ± 10.4	108.3 ± 4.0	89.8 ± 8.0	175.1 ± 14.8	141.0 ± 2.5
1.95	123.6 ± 0.4	132.2 ± 1.6	154.3 ± 11.2	183.4 ± 3.6	180.1 ± 6.8	194.7 ± 1.1	173.3 ± 2.6	185.4 ± 12.8	138.1 ± 2.2	112.7 ± 10.8	183.7 ± 6.9	159.6 ± 4.9

DCM-dichloromethane, DEE-diethyl ether, EA-ethyl acetate.

Supplementary Table S7. SARS-CoV-2 Mpro activity in serial dilution of isolated compounds. The results were presented as SARS-CoV-2 Mpro inhibition [%].

	**1**	**2**	**5**	**10**	**11**
Concentr. [µM]	Vanicoside A	Vanicoside B	Emodin	Procyanidin C1	Procyanidin B2 3,3'-di-O-gallate
100	70.7	63.80	51.50	22.30	36.70
66.7	64.8	48.20	27.50	0	1.00
44.4	57.7	34.10	19.50	0	0
29.6	47.7	29.20	12.50	0	0
19.8	26.3	2.20	0.10	0	0
13.2	8.2	0	0	0	0
8.78	0	0	0	0	0
5.85	0	0	0	0	0
3.90	0	0	0	0	0

Supplementary Table S8. SARS-CoV-2 Mpro activity in serial dilution of extracts and fractions from R. japonica (R.j.) and R.sachalinensis (R.s.) rhizomes. The results were presented as SARS-CoV-2 Mpro inhibition [%].

	**12**	**13**	**14**	**15**	**16**	**17**	**18**	**19**	**20**	**21**	**22**	**23**
Concentr. [μg/ml]	*R.j.* acetone	*R.s.* acetone	*R.j.* DCM	*R.s.* DCM	*R.j.* DEE	*R.s.* DEE	*R.j.* EA	*R.s.* EA	*R.j.* butanol	*R.s.* butanol	*R.j.* water	*R.s.* water
50	83.6	93.4	65.5	78.4	51	52.4	79.1	61.2	91	97.4	61.9	89.3
33.3	73.5	91.6	43.1	59	16.6	8.9	51	56.8	87.5	93.6	46.1	81.6
22.2	58.5	85.2	29.8	45	0	0	19	31.1	79.2	90.6	15.6	66.3
14.8	34.2	71.2	11.5	32.7	0	0	0	4.5	66.4	81	0	48.5
9.88	12.9	50.6	0	5	0	0	0	0	57	68.4	0	33.7
6.58	0	21.7	0	0	0	0	0	0	36.6	57.1	0	4.8
4.39	0	1.6	0	0	0	0	0	0	8.6	39.2	0	0
2.93	0	0	0	0	0	0	0	0	0	10.2	0	0
1.95	0	0	0	0	0	0	0	0	0	0	0	0

DCM-dichloromethane, DEE-diethyl ether, EA-ethyl acetate.
